# Recent Progress in Gels for Neuropathic Pain

**DOI:** 10.3390/gels9050417

**Published:** 2023-05-16

**Authors:** Ciprian Pușcașu, Anca Zanfirescu, Simona Negreș

**Affiliations:** Faculty of Pharmacy, “Carol Davila” University of Medicine and Pharmacy, Traian Vuia 6, 020956 Bucharest, Romania; ciprian.puscasu@umfcd.ro (C.P.); simona.negres@umfcd.ro (S.N.)

**Keywords:** neuropathic pain, tramadol, capsaicin, cubosomes, niosomes, pregabalin, gabapentin

## Abstract

Neuropathic pain is a complex and debilitating condition that affects millions of people worldwide. While several treatment options are available, they often have limited efficacy and are associated with adverse effects. In recent years, gels have emerged as a promising option for the treatment of neuropathic pain. Inclusion of various nanocarriers, such as cubosomes and niosomes, into gels results in pharmaceutical forms with higher drug stability and increased drug penetration into tissues compared to products currently marketed for the treatment of neuropathic pain. Furthermore, these compounds usually provide sustained drug release and are biocompatible and biodegradable, which makes them a safe option for drug delivery. The purpose of this narrative review was to provide a comprehensive analysis of the current state of the field and identify potential directions for future research in the development of effective and safe gels for the treatment of neuropathic pain, ultimately improving the quality of life for patients suffering from neuropathic pain.

## 1. Introduction

Neuropathic pain (NeP) is caused by damage or dysfunction of the nervous system, which can result in abnormal sensory processing and pain signals being sent to the brain [1]. Its etiology is complex, involving nerve damage or injury (owing to trauma, surgery, infection, or diseases, such as diabetes, multiple sclerosis, or HIV), compression or entrapment of nerves (e.g., in carpal tunnel syndrome or herniated discs), and diseases affecting the nervous system (as in stroke, spinal cord injury, or Parkinson’s disease) [2].

It may also be the result of exposure to chemotherapy or radiation therapy or of the administration of certain medications (e.g., antivirals or anticonvulsants) [3]. Independent of the etiology, negative (deficits of different somatosensory qualities) and positive (paresthesia and dysesthesia, paroxysmal pain, and ongoing superficial pain) sensory symptoms coexist in NeP [4]. Stimulus-evoked symptoms such as hyperalgesia and allodynia may occur in addition to spontaneous pain and are rarely the only pain manifestation [4].

An increasing trend in the prevalence of NeP is reported: 10% in 2017 vs. 5% in 2008, in the US [5]. Furthermore, a recent study approximated that about 15% of nursing home residents in the US suffer from NeP [6]. A recent research showed that the aged-standardized prevalence of chronic polyneuropathy is 3.3% for the European Union, 3.0% for the United States, and 2.3% for the world population and is expected to increase by ±25% in the next 20 years based on the expected age distributions [7]. Furthermore, in the young population, the incidence of diabetic neuropathy ranges from 2.6% to 11% and cardiac autonomic neuropathy from 4% to 39% of the patients with type 2 diabetes. Thus, diabetic neuropathy and cardiac autonomic neuropathy are the most common forms of neuropathy in adolescents and young adults [8].

The symptoms of NeP can be debilitating and have a significant impact on a person’s quality of life [4]. In patients with chronic neuropathic pain, physical and mental health declines more than in those with other types of chronic pain [9,10]. This highlights the impact of neuropathic pain’s nature on the quality of life and the need for complex therapy [11]. The costs of NeP are substantial and include medical expenses and productivity loss, adding to the economic burden [12].

Managing Nep can be frustrating and often represents a trial-and-error-based process [12]. Unlike other types of pain, neuropathic pain often does not respond well to traditional pain medications, such as opioids or nonsteroidal anti-inflammatory drugs. Even with the most effective treatment approaches, many individuals with neuropathic pain may experience ongoing symptoms and disability [12,13]. In one investigation, patients with NeP were more willing to take opioids and other pain treatments than those with chronic nonneuropathic pain, yet experienced less pain relief from medications [14]. Besides unsatisfactory effectiveness, even therapeutic agents used as first-line therapy of NeP, such as tricyclic antidepressants, selective inhibitors of serotonin and norepinephrine reuptake, and gabapentinoids, are associated with severe adverse effects. Hence, careful dosage is crucial, particularly in the elderly, as some patients experience adverse effects even with the lowest doses [15].

In the last decade, the use of analgesics-containing gels has gained attention over their oral administration due to several factors. Pregabalin, gabapentin, amitriptyline, and tramadol are among the agents most frequently used today as oral therapy and become the focus of research on developing new gels in the management of NeP [16]. Applied both topically or injectable, gels provide targeted and localized pain relief, allowing for the delivery of medication directly to the affected area—this is particularly beneficial for individuals with neuropathic pain, which is often localized to specific areas [17]. This targeted delivery can result in higher drug concentrations at the site of action, which can lead to improved efficacy and reduced systemic side effects compared to oral administration [18]. The use of new gels can provide sustained drug release over a longer period of time, allowing for more continuous pain relief without the need for frequent dosing [18]. As a result, the use of analgesic-containing gels has emerged as a promising alternative to oral administration for individuals seeking targeted pain relief with fewer systemic side effects [16]. 

Despite the development of such gels with targeted and sustained drug release and potentially effective in NeP, in the last decade, no recent review summarizing their characteristics exists. Therefore, we aimed to summarize effectively the novelties and improvements made in the field of research of gels destined to treat NeP, focusing on how a certain formulation can influence drug release, bioavailability, and skin permeation, factors which can significantly impact the effectiveness and safety of the medication.

## 2. Pharmacological Treatment of Neuropathic Pain

Epidemiological surveys indicate that numerous patients with neuropathic pain receive improper treatment [19], owing to inaccurate diagnosis, limited understanding of pain mechanisms, and drugs with poor effectiveness.

A first-line therapy recommended by the guidelines of the European Federation of Neurological Societies (EFNS) [20], the International Association for the Study of Pain (IASP) [21], and the American Academy of Neurology [22] consists in orally administered tricyclic antidepressants, amitriptyline (25–150 mg/day), gabapentin (1200–3600 mg/day), and pregabalin (150–600 mg/day) for various types of painful neuropathy (excluding trigeminal neuralgia) [23].

The mechanism of action of amitriptyline is complex [19]. Besides blocking Na^+^, K^+^, and Ca^2+^ voltage-gated ion channels, amitriptyline also blocks cholinergic, muscarinic, α2-adrenergic, nicotinic, histaminergic, opioid, adenosine, and N-methyl-D-aspartate receptor channels. The main side effects include dry mouth, weight gain, drowsiness, cognitive impairment, high cardiotoxicity [24], walking disturbances, and falls [25,26].

Gabapentin and pregabalin are used mostly in the management of diabetes-induced peripheral neuropathy and postherpetic neuralgia. The analgesic effect of gabapentin arises from increased GABA synthesis, α2δ subunit binding of voltage-gated calcium channels, and non-NMDA receptor antagonism. Gabapentin’s adverse reactions occur rather frequently (10–20%) and include somnolence, dizziness, ataxia, and fatigue [26]. Pregabalin reduces the release of neurotransmitters in a manner similar to gabapentin, namely by binding to the α-2-delta subunit of calcium channels [27]. When administered orally, pregabalin has side effects, such as dizziness, drowsiness, dry mouth, blurred vision, poor concentration, decreased levels of thrombocytes, and hypersensitivity [28].

Due to their established efficacy, serotonin and norepinephrine reuptake inhibitors (SNRIs), which block the reabsorption of the neurotransmitters serotonin and norepinephrine in the brain, can also be currently recommended (duloxetine 60–120 mg/day, venlafaxine 150–225 mg/day) as first-line treatment in painful polyneuropathy [22]. SNRIs are mostly recommended in the treatment of peripheral diabetic neuropathy, fibromyalgia, and back pain [23]. Possible adverse effects of SNRI treatment include sickness, dry mouth, vertigo, headache, and enhanced sweating [19]. However, their most severe adverse effects include suicidal thoughts and behavior, hypertension, bleeding, severe hyponatremia, and serotonin syndrome [29]. In addition, IASP guidelines include gabapentin extended-release and gabapentin enacarbil as first-line therapies [21]. 

Lidocaine is a local anesthetic medication that has been used in the treatment of neuropathic pain. When lidocaine is administered topically or injected into the affected area, it can block the transmission of pain signals from the peripheral nerves to the brain and can provide relief for several hours. It is often used to treat localized neuropathic pain, including postherpetic neuralgia, diabetic neuropathy, and complex regional pain syndrome. It is applied directly to the affected area. Topical lidocaine patches (up to 3 patches/day) are recommended in postherpetic neuralgia, especially in the elderly, as first-line therapy in EFNS guidelines [20] and second-line therapy according to IASP guidelines which consider the quality of evidence supporting the use of lidocaine is weak [21,30]. However, the therapeutic potential of lidocaine patches is modest and side effects are limited to applied areas [31]. Additionally, the American Academy of Neurology included sodium channel blockers for the treatment of neuropathic pain as they demonstrated their efficacy in reducing pain in meta-analyses [22].

Tramadol (200–400 mg/day) and capsaicin cream are used as second-line therapy in all therapy guidelines [22]. Tramadol has a mixt mechanism being an opioid weak agonist and an SNRI and is effective in neuropathic pain [31]. This drug can induce somnolence and confusion in adults, but its advantage compared to potent opioids is the lower risk of abuse [32]. 

Capsaicin patches (8%) have high-quality evidence and showed prolonged efficacy for approximately 3 months in diabetic or nondiabetic neuropathies [30]. As a natural alkaloid, capsaicin is the active ingredient in the dried fruit or ground powder (paprika) of hot peppers and acts by selectively binding to TRPV1 expressed on Aδ and C fibers [33]. It is systemically absorbed in a low percentage, and the side effects are manifested by transient skin reactions, such as pain, itching, and redness [30]. These side effects together with the potential safety concerns on sensation with long-term use exist, which led to their use as second-line therapy [31]. 

IASP and EFNS guidelines recommend the use of strong opioids only as a third-line therapy [20]. Despite being extremely effective, the use of these therapeutic agents is limited owing to their high risk of abuse [26] and a recent upward trend in the prescription of opioids causing overdose death and other opioid-related morbidities, particularly in the US, Canada, and the UK [28,29,30]. Furthermore, the American guide discourages the use of opioids considering them to be nonsuperior to nonopioid medications [31].

Subcutaneous botulinum toxin type A is used as the final therapeutic alternative in refractory cases, because its administration is painful, and long-term effects are limited [28]. Botulinum neurotoxins bind to neuronal cholinergic receptors [32], acting at alpha as well as gamma motor endings and altering spinal as well as cortical motor mechanisms [34]. Botulinum toxin type A, given subcutaneously in doses of 100–300 IU, requires administration in specialized units. Due to painful administration, local anesthetics and 50% nitrous oxide inhalation are recommended before and during treatment to decrease the pain [30].

Drug combinations (gabapentin/opioids or gabapentin/tricyclic antidepressants) are recommended to those patients who are partial responders to analgesic monotherapy [24]. Furthermore, for the first time, EFNS recommends cannabinoids in neuropathic pain in refractory cases [20].

Even though individual agents within the TCA (tricyclic antidepressants), SNRI, and gabapentinoid groups have similar efficacy in neuropathic pain, their adverse effect profile is different [32]. Patients with preexisting constipation, urinary retention, or orthostatic hypotension may show a lower tolerability for TCAs. In addition, cognitive impairment, gait disturbances, and falls may occur, especially in the elderly population [27,34]. On the other hand, the side effects of SNRIs and sodium channel blockers, such as nausea, tiredness, and vertigo, may be intolerable for patients with similar preexisting symptoms [27]. Patients with comorbidities causing peripheral edema should cautiously use gabapentinoids as they can also induce peripheral edema [27]. 

The average duration for a treatment to show efficacy is approximately 12 weeks. Neuropathic pain treatment should be administered for at least 12 weeks before being evaluated and considered ineffective, appropriate, or intolerable [27]. Regardless of each medication’s unique side effect profile, vertigo, somnolence, and tiredness are common in oral therapeutic regimens, while application site reactions occur in topical medication. A summary of the pharmacological therapeutic options is given in Table 1.

Considering that the therapeutic options for treating neuropathic pain have limited effectiveness in relieving pain and numerous side effects, few drugs with novel mechanisms of action are under clinical development for the treatment of this pathology [35], including selective sodium blocking agents such as Nav1.7 antagonists [36], angiotensin type II (EMA) [37], TPRV1, and nerve growth factor antagonists [35]. However, it will take some time before new therapies will be available.

Therefore, developing topical preparations, such as gels, containing substances known to be effective against NeP represents an attractive option as they would provide a faster onset of action, targeted delivery, improved adherence, a lower risk of gastrointestinal side effects, flexibility in dosing, and reduced drug interactions.

## 3. Development of Analgesic-Containing Gels Useful for NeP

Over the past decade, several types of gels containing analgesics for NeP therapy have been developed, based on the properties of the active substances for drug delivery applications (Table 2). Such gels are based on natural polymers such as chitosan or gelatin, which provide excellent biocompatibility and biodegradability [38]. These gels can be cross-linked with a variety of agents, such as glutaraldehyde, genipin, or sodium tripolyphosphate, to improve their mechanical and swelling properties [39]. Another type of hydrogel is based on synthetic polymers, such as polyethylene glycol or polyvinyl alcohol, which provide tunable properties and controlled drug release capabilities [40]. Furthermore, they can be modified with various functional groups to enhance their stability and biocompatibility. The incorporation of the active substances into these hydrogels can be achieved by different methods, such as physical entrapment, covalent bonding, or electrostatic interactions.

Understanding and optimizing these factors can improve the overall effectiveness of such treatments. Some of these preparations have shown promising analgesic effects after transdermal or subcutaneous delivery in animals, and thus, they might offer an improved therapeutic outcome in NeP.

### 3.1. Capsaicin

In clinical practice, capsaicin is primarily used as a topical analgesic to treat neuropathic pain. Capsaicin has also been studied for its potential therapeutic effects in other conditions, including osteoarthritis, psoriasis, gastritis, and migraines, although more research is needed to determine its efficacy and safety in these areas [64]. 

Regarding the currently authorized products containing capsaicin, they include prescription patches of 8%. Usually, transdermal capsaicin implies the use of up to 4 patches for 30–60 min once every 3 months. Overuse of capsaicin patches can lead to severe skin irritation or other side effects. In vitro data demonstrate that the release rate of capsaicin from patches is linear over the duration of application, and approximately 1% of the amount of capsaicin is absorbed into the layers of the epidermis and dermis during an 1-hour application [65]. Several creams containing capsaicin exist worldwide as supplements [66]. Capsaicin cream, available as over-the-counter products with 0.025–0.1% concentrations, with 0.1% considered high potency, can be applied up to four times a day. Although burning, stinging, or itching may occur, these sensations diminish over time [67].

Capsaicin-containing gels have been extensively studied in the last decade. Most of the studies used natural polymers such as chitosan [68], alginate [69], or gelatin [70] to develop capsaicin-containing hydrogels. Several synthetic polymers, including polyethylene glycol (PEG) and polyvinyl alcohol (PVA), were also used. The incorporation of capsaicin into the hydrogels was achieved by different methods, such as physical mixing and more recently, by encapsulation. The release profile of capsaicin from the gels varied depending on the type of polymer and the method of incorporation.

Older studies investigated the formulation of hydrogels containing capsaicin through physical mixing. Wang et al. [71] investigated the in vitro and in vivo skin absorption of capsaicin from chitosan/carboxymethylcellulose-based hydrogels and compared it with that of commercialized creams containing capsaicin. Drug partition between the skin and the gel matrix was critical in the permeation process. As expected, the in vitro permeation of capsaicin from the tested gels is depended on the physicochemical nature and the concentration of the polymer used. Thus, adding the nonionic polymer Pluronic (Plo) F-127 to the gels produced a delayed release of capsaicin. On the other hand, a higher capsaicin permeation rate was obtained in in vitro studies with cationic chitosan and anionic carboxymethyl cellulose hydrogels than with cream bases. In a dose-dependent manner, the cream produced skin erythema in vivo. However, the dose-dependence was not observed in gels which had a lower irritative effect than commercially available cream [71]. 

Peng et al. [72] designed and investigated the transdermal controlled release of cubic phase gels containing capsaicin. Release studies demonstrated that cubic phase gels imprint a sustained system for capsaicin, the release rate being affected by the initial water content, distribution of capsaicin in the lipid bilayers, and cubic phase gel swelling.

Capsaicin-loaded 1% nanolipoidal carriers (NLCs) were designed to increase permeation and provide analgesic and anti-inflammatory effects with reduced skin irritation [73]. These NLCs and gels with capsaicin-loaded NLCs demonstrated sustained release, noncytotoxic properties, enhanced penetration, and improved pain threshold in a dose-dependent manner, while inhibiting inflammation more effectively than conventional preparations. Reduced skin irritation suggests NLCs as a potential carrier for topical delivery of capsaicin in pain and inflammation therapy [73]. 

Aylang et al. [74] extracted a natural β-chitin–protein complex film from waste shells of *Ensis* spp. After production and physicochemical characterization of the film, capsaicin was loaded. The loading capacity was 5.79%, and over a period of 120 h capsaicin remained stable with a sustained release rate. Maximum release of capsaicin was recorded as 50.49% (48 h) for pH 4.0, 59.81% (72 h) for pH 5.5, and 59.02% (96 h) for pH 7.4.

Another study developed and characterized a chitosan-based hydrogel containing capsaicinoids-loaded nanocapsules for topical delivery. Several chitosan hydrogels were prepared to determine the optimal composition. The most suitable gel contained the lowest amount of lactic acid (1.5%) and an intermediate amount of chitosan (3.5%), ensuring a pH of 4.34 ± 0.11. After 30 days of storage, the gel exhibited a slight increase in consistency and a decrease in the flow index and pH [68].

Peng et al. used phytantriol- and GMO-based cubosomes as a targeted, sustained transdermal delivery system for capsaicin [75]. Their skin retention of capsaicin (4.32 ± 0.13 μg) was higher than that of capsaicin cream (0.72 ± 0.13 μg). The cubosomes were stable under strong light and high temperatures for up to 10 days and caused minimal irritation. Phytantriol-containing cubosomes exhibited higher permeation and more sustained skin retention of the drug than GMO-based cubosomes and the cream.

To conclude, the newly synthesized gels showed a release rate ranging from 12.96% to 81% of capsaicin and improved skin permeation of the drug. Furthermore, the encapsulation of capsaicin in nanocarriers reduces the irritative effect.

### 3.2. Tramadol

The physicochemical characteristics of tramadol presented in Table 1 make it suitable for incorporation in gels. 

#### 3.2.1. Gels for Transdermal Use

Shah et al. [18] investigated the possibility of transdermal delivery of tramadol using a proniosomes-based gel formulation and evaluated its therapeutic potential in vivo. Surfactant-based colloidal drug carriers such as niosomes and their hybrids (proniosomes) effectively transport larger drug quantities to the skin, enabling controlled release for systemic absorption [76]. Dry proniosomes formulations are converted to niosomes after the hydration [76]. For example, the proniosomes embedded in a suitable gel form niosomes by absorbing water [18]. Niosomes increase drug permeation as they overcome the barrier properties of skin and form a drug depot [77]. The formula with the best drug release, stability, and transdermal efficacy contained 100 mg tramadol hydrochloride, 1800 mg Span 80, 1800 mg lecithin, and 200 mg cholesterol [18]

Natori et al. [78] developed a tramadol-containing hydrogel film composed of 20% (*w*/*w*) hydroxypropyl methylcellulose (HPMC), which was obtained by irradiation with electron beams. This formula presented similar transparency and elasticity as commercially available dressings. Various electron beam doses imprinted differences in release and permeation rate from hydrogel films containing tramadol; thus, hydrogel films irradiated at 50 kGy showed enhanced release than those irradiated at 30 kGy.

For effective pain management, tramadol-hydrochloride-encapsulated transethosomes were formulated by cold method with different lipoidal carrier systems. A total of 12 formulations were prepared, formulations being designed by using ethanol, edge activator (Span 20 and Cremophor EL-35), and phospholipids (soya lecithin, l-α phosphatidylcholine from egg yolk). The highest encapsulation efficiency was observed for the transethosomes obtained using Cremophor EL-35 0.5% as an edge activator. Their average size ranged between 149.34 and 198.10 nm. As the concentration of edge activator in the formulation increased, the vesicular size decreased. Lecithin-based formulations exhibited a higher particle size and higher viscosity [79]. 

#### 3.2.2. Gels for Parenteral Use

Hydrogels have emerged as a promising material for parenteral drug delivery due to their ability to absorb large quantities of water while maintaining a three-dimensional network structure. The highly hydrated nature of hydrogels allows them to mimic the extracellular matrix of tissues, promoting biocompatibility and reducing immune reactions [80]. Additionally, hydrogels can be created to respond to various stimuli, such as pH, temperature, and enzymatic activity, enabling controlled drug release [81]. Moreover, the unique physicochemical properties of hydrogels, such as their high water content and tunable mechanical properties, make them ideal candidates for encapsulating and delivering a wide range of therapeutics, including tramadol [82]. 

Barati et al. [83] developed a chitosan-based thermoresponsive in situ gel-forming formulation with tramadol purposive for subcutaneous injection. Their clear advantage, compared to oral formulations, consists in providing a depot for slow release of the drug over an extended period of time at the site of the injection over 8 h. Furthermore, adding pentasodium triphosphate (TPP) to the formula resulted in the formation of spherical nanocavities in the homogenous containing gel structure, resulting in a higher percentage of the cumulative release than the formula without TPP. The nanostructures were not present in the sol state. They appeared when the sol–gel transition occurred, leading to the emergence of a new concept: pro-nanogels.

Dos Santos et al. studied PL-based binary hydrogels composed of PL 407 and PL 188 [82]. A minimal PL concentration of 35% formed thermo-reversible gels. Drug–micelle interaction studies showed PL 407–PL 188 binary systems with drug partitioning into micelles. The presence of tramadol hydrochloride increased enthalpy variation values during the sol–gel transition phase. Rapid hydrogel dissolution reached 80–90% in 24 h. Tramadol incorporation into the binary system prolonged analgesic effects, extending release for 48–72 h after subcutaneous injection.

### 3.3. Gabapentin

Topical administration of gabapentin is associated with minor adverse effects [84]. Therefore, topical formulations are being developed. However, gabapentin has some physicochemical characteristics that may make it difficult to incorporate into gels, as shown in Table 1 [44]. 

Despite challenges, successful incorporation of gabapentin into gels has been reported. One clinical trial reported that a 6% gabapentin cream effectively ameliorates vulvodynia [85]. A 10% *w*/*w* topical gabapentin gel applied thrice daily significantly reduced allodynia and hyperalgesia in a rat sciatic nerve constriction model without motor impairment [86]. The same pharmaceutical form also attenuated cisplatin-induced neuropathic allodynia and heat-hypoalgesia [87].

Martin et al. [88] prepared and evaluated several formulations of gabapentin, including preformulated oil-in-water bases and Carbopol-based hydrogels with permeation enhancers. To prevent crystallization, a maximum of 6% (*w*/*w*) gabapentin was incorporated within all Carbopol^®^ hydrogels. The 5% (*w*/*w*) DMSO Carbopol^®^ gels were stable for at least 3 months under ambient conditions. 

A new gabapentin formulation was recently developed—chitosan-g-poly(acrylic acid-co-acrylamide) hydrogel composite—containing gabapentin and evaluated at different pH, temperature, and time intervals for drug delivery and controlled release of gabapentin. The research showed a maximum encapsulation of 64% and a drug loading efficiency of the hydrogel of 71% and that the formula imprinted a sustained-release manner of the drug from the hydrogel. In the first 2 h, 90% of the total gabapentin was released according to the dual temperature and pH-responsive hydrogel composite release study [89].

Shakshuki et al. [90] compared three different forms containing gabapentin: Lipoderm cream, Versabase gel, and Emollient cream. All these three forms contained 10% gabapentin. 

At 25 °C and 40 °C, the potency of gabapentin in Lipoderm cream highly increased after 28 and 90 days, respectively. In contrast, gabapentin has deteriorated in Emollient cream. At 25 °C, the drug combined with Lipoderm cream did not show changes in organoleptic properties for up to 28 days, but physical changes were observed in other bases. Gabapentin was recrystallized from Versabase gel and Emollient cream within 14 days.

Another study formulated and characterized gabapentin-encapsulated elastic liposomes and compared their efficiency in transdermal delivery of gabapentin with that of the compounded gabapentin-based Plo lecithin organogel. Gabapentin liposomes-containing gel had a significantly slower release rate compared to the Plo lecithin organogel (12 h vs. 4 h). Moreover, after 24 h, liposomes highly increased the percutaneous penetration of gabapentin through the porcine skin leading to greater concomitant drug concentrations compared to the Plo lecithin organogel [91]. 

### 3.4. Pregabalin

Currently, there are no authorized topical formulas containing pregabalin. Oral formula has side effects which include dizziness, sleepiness, dry mouth, and blurred vision, among others [92]. Topical formulas could reduce systemic drug exposure and penetration into the brain, minimizing the occurrence of CNS-mediated side effects [93].

Four pregabalin preparations for transdermal application were developed in a recent study [94]: 0.4% aqueous solution, Plo lecithin organogel, hydrophilic cream, and lipophilic cream. The organogel had the highest permeability, followed by the aqueous solution, while creams showed no permeation. Pregabalin was distributed into the dermis 1 h after the application of the organogel. Furthermore, in vivo testing using a mouse model of diabetic neuropathy demonstrated that only the organogel had a significant analgesic effect. This study demonstrated for the first time that pregabalin reached the dermis following topical application of a Plo lecithin-based organogel formulation.

Arafa et al. [92] prepared mucoadhesive topical gels with pregabalin alone or encapsulated in niosomes. Higher cholesterol ratios increased pregabalin entrapment efficiency, as increasing the concentration of cholesterol inhibits the conversion of the gel into liquid and enhances the encapsulation of hydrophilic drugs. Furthermore, the formula with the highest cholesterol content showed the lowest percent of drug release. HPMC and carbopol hydrogel formulations showed a higher release rate (91.2 ± 0.05%) of PG compared to niosomal PG formulations. 

Cevik et al. [95] reported a novel method for synthesizing pH-responsive composite hydrogels using visible light. The pH-responsive layer is formed using poly(methacrylic acid-g-ethylene glycol) [P(MAA-g-EG)] as the macromer, eosin Y as the photoinitiator, and triethanolamine as the co-initiator. The three types of hydrogels, plain, [P(MAA-g-EG)], and P(MAA-g-EG) hydrogels, varied with the composition of the hydrogel prepolymer and the photoinitiation mechanism, with those formed under visible light preserving their integrity better than the ones formed under UV light. Therefore, cross-linked styrene–butadiene–styrene particles enhance the integrity of the hydrogel. Furthermore, in vitro fibroblast viability assay and in vivo implantation experiments indicated the hydrogels were nontoxic and nonirritant. 

Another study evaluated different formulas of emulgels-containing pregabalin using Carbopol 940 and other polymers. The best drug release rate was achieved with Carbopol 940 0.4% and HPMC K15M 0.4%, offering rapid analgesic effects while avoiding pregabalin’s CNS-mediated side effects [93].

Thus, optimized gel formulations containing pregabalin resulted in a significant release rate of up to 90% and an improved skin permeation for up to 240 h.

### 3.5. Amitriptyline

Oral amitriptyline can cause serious side effects [50]; therefore, developing topical formulas with amitriptyline for treating NeP is of great interest. However, no registered topical formulas exist currently. This may be due to the inconsistent results seen in clinical studies. Several clinical studies reported no analgesic efficacy of amitriptyline in patients with neuropathy when topically administered as 5% or lower concentration formulas [96]. Furthermore, most of these studies do not report the used formulation. Ho et al. [97] reported the use of a Plo lecithin organogel formulation. Despite reducing pain intensity, topical lidocaine induced minimal clinical improvement, whereas placebo and topical amitriptyline were ineffective. No rationale was given to justify the use of Plo. However, studies using higher concentrations of amitriptyline (10%) reported a more consistent analgesic effect [19]. 

Conversely, Shakshuki et al. [98] evaluated amitriptyline hydrochloride (1%, 5%, and 10%) compounded with three different bases: Lipoderm base, Emollient Cream, and Mediflo 30 Plo lecithin organogel. Mean cumulative release after 24 h from the 10% formulation was significantly higher from the Mediflo Plo lecithin organogel than from the Lipoderm base or Emollient Cream: 53.2% vs. 23.9% and 41.8%, respectively. The authors had an extremely important observation: Amitriptyline released from formulas containing less than 1% of active substance did not permeate through the artificial skin membrane. A similar observation was made for the 5% Mediflo Plo lecithin organogel. Furthermore, despite having the highest amitriptyline release rate, 10% Mediflo Plo lecithin had the lowest permeation over 24 h. Amitriptyline 5% in Lipoderm base had the highest flux—this indicates that using a more lipophilic base may be a more suitable choice for creating topical amitriptyline preparations due to its high lipid content and lipophilic properties. By containing a high percentage of lipids, such as isopropyl myristate and caprylic/capric triglyceride, which are highly lipophilic, Lipoderm can improve the solubility and permeability of lipophilic drugs [99]. Another suggestion would be using a pH adjuster. Amitriptyline is a weak base, and its solubility increases at acidic pH. Thus, an appropriate pH adjuster may be used to adjust the pH of the gel to a range where amitriptyline is more soluble.

### 3.6. Other Substances

Despite not being included in the therapy guidelines for NeP, some reports indicate that the use of topical preparations containing other substances may be useful in the treatment of pain. 

Ketamine is primarily used for anesthesia and pain relief. In recent years, it has also gained interest as a treatment for depression, posttraumatic stress disorder, and other psychiatric conditions. The mechanism of action of ketamine is complex and not yet fully understood. It is an N-methyl-D-aspartate receptor antagonist, and it seems it can also act on other receptors in the brain, such as the dopamine and serotonin receptors. Ketamine is typically administered intravenously, and its use is associated with several side effects, including dissociative effects, hallucinations, and changes in blood pressure and heart rate. Therefore, topical formulations may be a great alternative [52]. Wang et al. [52] developed a ketamine-loaded PL F127 stabilized reduced graphene oxide hydrogel for sustained transdermal drug delivery. Adding Plo F127 stabilized reduced graphene oxide sustaining the release of ketamine due to unique π-π stacking interaction. The ex vivo release study showed sustained release of ketamine from hydrogel compared to the control hydrogel, consistent with the results of the in vivo tail-flick evaluation, in which ketamine-loaded reduced graphene oxide had a significantly prolonged analgesic effect (24 h) compared to the ketamine control hydrogel (4 h). Baclofen interacts with two types of receptors, gamma amino butyric acid A and B, inhibiting chemokine-induced chemotaxis [100]. Adverse effects following oral administration include muscle weakness, nausea, somnolence, and paresthesia and affect between 25% and 75% of patients [56]. 

In a study, a topical gel containing baclofen niosomes was developed by adjusting the ratios between various nonionic surfactants (Span 60, 40), cholesterol, and charge-inducing agents. The entrapment efficiency of the formulations ranged from 4.37% to 80.31%. Encapsulating the drug into niosomes allowed sustained and controlled drug release. The incorporation of free baclofen and two niosomal baclofen formulations into Carbopol 934 led to faster permeation than Pluronic F127. In vivo examination revealed no significant analgesic differences between baclofen niosomal gel and marketed baclofen tablets after 24 h [100]. 

Baclofen-containing hydrogels with controlled release were prepared using different hydrophilic polymers. A total of 18 formulations were prepared and evaluated. The topical baclofen gels showed good physical properties, and the optimized formulations were stable at room temperature [101]. 

Another study formulated baclofen-loaded Eudragit^®^ RL100 nanoparticles by nanoprecipitation method. After characterization, particle size decreased with increasing Eudragit^®^ RL100, and the smallest size was observed in formulas containing an organic phase (acetone: methanol) in a 1:3 ratio. The highest encapsulation efficiency occurred with the highest baclofen-loaded Eudragit^®^ RL100 concentration and the same organic phase ratio [102]. 

Topically administered nonsteroidal anti-inflammatory drugs reduce proinflammatory PGs by inhibiting cyclooxygenase COX-2, interfering with the nociceptive pathway [103]. Conventional topical preparations are not effective in NeP. However, newer formulations have optimized characteristics. Furthermore, long-term oral administration of NSAIDs can cause gastrointestinal symptoms ranging from vague complaints to duodenal ulcer symptoms.

Naproxen was investigated in a formula using glycofurol as a vehicle-based gel and three various gelling agents (Carbopol 974P, Gantrez AN 119, and polyvinylpyrollidone K30). Skin permeation rates and lag times were evaluated to obtain the best gel formulation. Permeability parameters, steady-state flux, permeability coefficient, and penetration index, showed an increase in optimized formulation containing 2% Transcutol as permeation enhancer. On the other hand, optimized novel glycofurol-based gel formulation showed no topical adverse effects in the skin irritation test. Glycofurol-based gel seems to ensure dermal and transdermal delivery of naproxen and may be used for water-insoluble drugs [104].

As a new approach for delivering therapeutic agents, researchers developed supramolecular hydrogels using small peptides conjugated with NSAIDS. The conjugation did not disrupt the binding of naproxen to COX-2. The presence of D-tyrosine on the D-peptide improved the activity and selectivity of naproxen no matter where the position of naproxen was on the side chain. Furthermore, the conjugation of naproxen greatly reduced its binding to COX-1, which in theory, would diminish the associated adverse gastrointestinal and renal effects [105].

Tryptophan N-capped dipeptides with naproxen were prepared using C-terminal dehydroamino acids. The hydrogels consisted of networks of micro/nanosized fibers formed by peptide self-assembly driven by aromatic group stacking interactions. Hydrophobic peptides (containing C-terminal dehydrophenylalanine) formed more elastic gels at lower critical gelation concentrations and revealed irreversible breakup, while gels with C-terminal dehydroaminobutyric acid and dehydroalanine showed structural recovery and partial healing of the elastic properties. Therefore, these hydrogels are promising drug–nanocarrier candidates [106].

A topical microemulsion-based hydrogel containing ibuprofen was prepared and evaluated. The optimum formulation contained 3% ibuprofen, 6% ethyl oleate, 30% Tween 80/PG (2:1), and water and showed a high permeation rate of 38.06 μg cm^−2^ h^−1^ in vitro using porcine skins. Xanthan gum was used as a gel matrix, for increasing viscosity. The studied microemulsion-based hydrogel presented good stability, being a promising vehicle for topical delivery of ibuprofen [107].

Mauri et al. evaluated the covalent tethering of ibuprofen to a hydrogel matrix via esterification. The COX inhibitory activity of ibuprofen was not affected after the modification of the terminal carboxyl group, ensuring a therapeutic effect that is comparable to that of its salt form. As a result of chemical functionalization, ibuprofen can be given in the form of its free carboxylic acid instead of its sodium salt, which provides a viable alternative. The free carboxylic acid form of ibuprofen is more soluble and diffuses more quickly than the salt form. By incorporating an ibuprofen-diol derivative into the hydrogel formulation, a more sustained release profile was achieved compared to the salt form. These ibuprofen-functionalized hydrogels could be used as injectable tools due to their sol–gel transition, which enables localized drug release and presents promising possibilities for in situ treatments [108]. 

Using chitosan, lipids, gum arabic, and polyvinyl alcohol, six formulations containing ibuprofen were prepared through ionic interaction, maturation, and freeze–thaw methods. The results showed that the lipid-conjugation-based hydrogel exhibited a higher conjugation efficiency and prolonged drug release than control. Furthermore, ibuprofen effectively reduced LPS-induced PGE2 synthesis [109].

Vivero et al. analyzed poly(hydroxyethyl methacrylate) hydrogels as nonsteroidal anti-inflammatory drugs’ delivery systems using 4-vinyl-pyridine and N-(3-aminopropyl) methacrylamide as cross-linkers. Incorporated monomers substantially increased ibuprofen (up to 10-fold) and diclofenac (up to 20-fold) loading, while drug release was limited (less than 10%) due to ionic/hydrophobic interactions. 

Water-swollen hydrogels transferred to pH 5.8 or 8.0 phosphate buffer or NaCl solutions showed release driven by competition with environmental ions, sustaining the release process for minimum 24 h for ibuprofen and almost 1 week for diclofenac owing to the remaining hydrophobic interactions and the high polymer density induced by poly(hydroxyethyl methacrylate) [110].

Another study analyzed hydrogel films loaded with ibuprofen using water-soluble polysaccharides such as cellulose sulfate, chitosan sodium, and tripolyphosphate via self-assembly method. The hydrogels formed a dense regularly shaped network due to polyelectrolyte complex formation via electrostatic interaction. Loading and encapsulation efficiency were 43.15 ± 4.88% and 60.65 ± 4.68%, respectively. The hydrogel films showed a sustained release profile during 1440 min test using mice skin [39]. 

### 3.7. Associations of Analgesic Substances

The combination of two or more therapeutic agents can be more effective considering the wide variety of pathophysiological mechanisms involved in the development and progression of neuropathic pain. A topical combination of baclofen, amitriptyline, and ketamine was evaluated in a double-blind, placebo-controlled trial, including patients with chemotherapy-induced peripheral neuropathy. The active formulation contained 40 mg of amitriptyline, 20 mg of ketamine, and 10 mg of baclofen in pluronic lecithin organogel and was applied twice a day for 4 weeks (a teaspoonful of gel to each affected area). Although improvements in sensory pain and motor scale were observed, they were not statistically significant, and no adverse effects or systemic toxicity were reported [96]. 

In an uncontrolled trial, Uzaraga et al. [111] assessed the efficacy of a topical treatment consisting in an amitriptyline 2%, ketamine 1%, and lidocaine 5% (AKL) containing gel in radiation-induced dermatitis and neuropathic pain. The gel was given to 60 individuals, three times per day until 2 weeks post-radiotherapy. A patient assessment was performed every 2–5 days during radiotherapy and at 2 and 6 weeks postradiotherapy. Pain was assessed using the University of Washington neuropathic pain scale. After AKL gel application, a reduction in pain intensity and other symptoms was recorded at 30 min and 2 weeks posttreatment compared to baseline, but fatigue and skin irritation occurred at the application site. 

A study case reported the effectiveness of the amitriptyline and ketamine containing gel in a patient with erythromelalgia. Over the next 2 weeks, the patient described “very promising results”, with a 60% decrease in pain intensity, primarily burning pain. This patient did not respond previously to oral paracetamol, NSAIDs, gabapentin, and amitriptyline [112]. These clinical trials support the idea of using topical preparations instead of oral therapy.

The method of preparation and the functional excipients used to obtain all these gels are presented in Table 3. As the local conditions within the tissue or cellular environment (pH, temperature) where the gel is applied can influence the efficacy and function of the gel, when available we also included such details in Table 3.

## 4. Conclusions and Perspectives

Management of NeP is often challenging and may require a multimodal approach involving early diagnosis, psychological therapy, as well as treatment to relieve symptomatic pain [113]. According to the latest guidelines, oral therapies are used as first-line therapies for the treatment of NeP [114], with topical therapies being the second line owing mostly to low efficacy. Furthermore, oral analgesics are often associated with high toxicity.

Gels represent a promising platform for transdermal drug delivery due to their unique physicochemical properties. Gels can provide a sustained drug release, improved bioavailability, and controlled delivery of drugs through the skin. The high water content of gels allows for better hydration of the stratum corneum, which can increase the permeability of the skin and enhance drug absorption [115]. In addition, gels can be designed to adhere to the skin, minimizing drug loss due to rubbing or sweat [116]. The gel matrix can also protect the drug from degradation and improve its stability during storage and transport [66]. Transdermal delivery systems prevent the occurrence of adverse effects by preventing peak and trough plasma concentrations and decreasing drug accumulation [67].

Over the years, several studies investigated the development of gels with capsaicine, amitryptiline, gapabentin, pregabalin, tramadol, and ketamine to be used in the treatment of NeP [117].

In recent years, different new forms of gels with therapeutic agents useful for NeP have been discovered and tested. Nanoemulsion containing capsaicin, proniosomes gel formulation containing tramadol, tramadol hydrochloride encapsulated transethosomes, chitosan-g-poly(acrylic acid-co-acrylamide) hydrogel composite containing gabapentin, pluronic lecithis organogel formulation containing pregabalin, Mediflo pluronic lecithin organogel containing amitriptyline, and ketamine-loaded Pluronic^®^ F127 stabilized reduced graphene oxide hydrogel for sustain drug delivery via transdermal route are among the pharmaceutical gels characterized, tested for the analgesic effect in vivo in preclinical or clinical studies and demonstrating a real potential to replace oral therapy. 

The recent developments made in the formulation of gels containing analgesics include:

- incorporation of vesicular carriers, such as niosomes, transethosomes, and cubosomes, with enhanced skin penetration and drug delivery compared to conventional liposomes [118]. These carriers protected the encapsulated drug from degradation and acted as local reservoirs of the drug. All the included studies revealed that the utilization of vesicular carriers as drug delivery systems enables a profound interaction with the skin layers, leading to sustained and efficient drug release for a wide range of drugs. However, a better understanding of this interaction would allow further optimization of the formulas.
- use of in situ gel-forming formulations which also ensured a prolonged and constant drug release. Furthermore, thermoresponsive polymers that can undergo a reversible sol–gel transition in response to temperature changes have been used in the preparation of injectable gels for drug delivery. In situ cross-linking was used as a trigger to stimulate this transformation.
- use of multicomponent gels that contain more than one type of polymer. They offer better mechanical stability and tunable properties.
- co-delivery of multiple drugs in a single gel formulation can improve treatment efficacy and reduce side effects.

Our investigation encountered several inherent limitations due to the heterogeneous nature of the study designs evaluated. For instance, disparate methodologies were employed to quantify drug release rates and skin permeation profiles, potentially leading to inconsistent results. Furthermore, although several gels were developed, they were not tested in preclinical or clinical studies. Thus, it is difficult to evaluate their real analgesic utility. We consider that creating uniform protocols of evaluation of drug release for gels and performing an appropriate preclinical and clinical assessment of these products would provide the very much needed scientific base for identifying new gels useful in the therapy of NeP.

In conclusion, recent advances in the formulation of analgesic gels have brought about significant improvements in drug delivery systems for pain management. The development of new gelling agents, additives, and drug delivery technologies has led to enhanced solubility, bioavailability, and controlled release of analgesics, resulting in better pain relief and improved patient compliance. Additionally, the development of personalized medicine and the exploration of nonopioid analgesics in gel formulations offer new directions for future research. While there are still challenges to be overcome, such as ensuring safety and addressing the need for targeted drug delivery, the progress made in recent years provides a strong foundation for the continued development of advanced analgesic gel formulations.

## Figures and Tables

**Table 1:** Pharmacological treatment of NeP.

Pharmacological Treatment of NeP						
First-Line Therapy			Second-Line Therapy		Third-Line Therapy	
TCA Amitriptyline	SNRI Duloxetine Venlafaxine	Gabapentinoids Pregabalin Gabapentin	Topical treatment Capsaicine Lidocaine	Tramadol	Strong opioids Morphine Oxycodone	Botulinum toxin type A
Painful neuropathy Nerve injury pain Postherpetic neuralgia Central postpartum pain Spinal cord injury	Diabetic neuropathy Fibromyalgia Back pain Diabetic pain	Spinal cord injury Herpetic neuralgia Phantom limb syndrome	Localized NeP: Postherpetic neuralgia Diabetic pain Regional pain syndrome Diabetic pain Central Pain	Chemotherapy-induced peripheral neuropathy Phantom limb syndrome	Diabetic pain Postherpetic neuralgia Central pain	Focal peripheral neuropathy and allodynia

**Table 2:** Pharmacokinetic and physicochemical characteristics of analgesic drugs used for neuropathic pain.

Substance	Absorbtion	Metabolism	Elimination	Half-life	Physicochemical Characteristics	References
Capsaicin	Oral administration 50 to 90% Intravenous/subcutaneous administration in the brain and spinal cord about 5 times higher than in blood; in the liver about 3 times higher than in the blood. Topical administration: rapidly and well absorbed through the skin.	Significant first-pass effect of liver after oral administration	Renal excretion	Following oral ingestion approximately 24.9 ± 5.0 min. Following topical application approximately 24 h	Low water solubility. pKa (Strongest Acidic): 9.93 pKa (Strongest Basic): −1.4 Melting point: 65 °C	[35,36,37]
Tramadol	Oral administration: rapidly and almost completely absorbed, with a bioavailability of 75%	Extensive first-pass metabolism in the liver	Primarily through metabolism by the liver; metabolites are excreted primarily by the kidneys.	5–6 h	Tramadol hydrochloride salt is highly water-soluble. The pH range of tramadol hydrochloride salt: 4.0–5.5. The melting point of tramadol: 180–184 °C. The solubility of tramadol in ethanol and chloroform makes it possible to incorporate it into hydro-alcoholic or lipophilic gel formulations.	[41,42]
Gabapentin	The oral bioavailability is inversely proportional to the administered dose.	Not appreciably metabolized	In the urine as unchanged drug.	5–7 h	pKa: 3.7 Octanol/water partition coefficient: 1.24, indicating that it is highly hydrophilic.	[43,44]
Pregabalin	Oral administration bioavailability ≥90% regardless of the dose	Less than 2% is metabolized	Almost exclusively eliminated in the urine.	6.3 h	Good water solubility. pKa: 4.2. Octanol/water partition coefficient: −1.35 Low molecular weight −159.23 g/mol	[45,46,47,48]
Amitriptyline	Rapidly absorbed following oral administration	Suffers from first-pass metabolism. The main active metabolite is nortriptyline.	Amitriptyline and its metabolites are mainly excreted in the urine.	About 25 h	Highly lipophilic, small-molecular-weight compound. Low water solubility: pKa: 9.4 Octanol/water partition coefficient: 3.3. High molecular weight: 313.87 g/mol.	[49,50]
Ketamine	Absorption is very rapid and the bioavailability is around 93%.	Mainly hepatic metabolism.	Manly urine excretion mainly in the form of metabolites	186 min	pKa: 7.5 Molecular mass: 238 g/mol Ketamine is typically administered intravenously, and its use is associated with several side effects.	[51,52,53]
Baclofen	Oral bioavailability: 70% to 85%	About 15% of the oral dose is metabolized in the liver.	70–80% is eliminated in an unchanged form by renal excretion.	2–6 h after oral administration.	Water solubility: 4 mg/mL pKa: 9.62 + 0.1 (amino group) 3.87 + 0.1 (carboxyl group) Adverse effects following oral administration affect between 25% and 75% of patients.	[54,55,56]
Naproxen	Peak plasma concentration after 1 h, peak plasma concentration for naproxen (free acid) is observed after 2 h	Heavily metabolized in the liver.	Approximately 95% of naproxen and its metabolites can be identified in the urine	12–17 h	Water solubility: 15.9 mg/L pKa: 4.15 Melting point: 152 °C	[57,58,59,60]
Ibuprofen	Very well absorbed orally.	Rapidly metabolized and biotransformed in the liver to the formation of major metabolites.	Eliminated in the urine.	1.2–2 h	Water solubility: 21 mg/L pKa: 5.3 Melting point: 75–77 °C	[61]
Diclofenac	Totally absorbed from the gastrointestinal tract.	Substantial first-pass metabolism	60–70% urinary elimination and 30% elimination through feces	2 h	Water solubility: 2.37 mg/mL pKa: 4.15 Melting point: 283–285 °C	[62,63]

**Table 3:** Analgesic-containing gels used for neuropathic pain.

Substance	Gel Type	Method of Preparation	Formula with Optimal In Vitro Parameters	Role of Agents	Parameter	Advantages of the New Formulation in the Treatments of Neuropathic Pain	References
Capsaicin	Hydrogel	Dissolution The certain quantity of polymer and drug was added into pH 4 buffer with continuous stirring for 1 h.	0.075% capsaicin, 6% CMC-Na	CMC, synthetic water-soluble cellulose added as matrix for drug delivery systems.	Rate of release 12.69 ± 0.58 g/cm^2^ per ½ h	Advantages The cumulative quantity of capsaicin 4 h after the application is bigger than that of marketed creams. CMC-Na showed better bio-adhesion to the skin, resulting in a prolonged time of location at the site of application and enhanced permeation efficacy. CMC-Na hydrogels can produce a fast onset and sustained duration of capsaicin release, in contrast to cream bases. Disadvantages All individuals revealed more powerful pungent sensation of CMC-Na hydrogels than cream bases.	[71]
Capsaicin	Biogel	Dissolution Capsaicin was weighed into the monooleate and heated to melt at 45 °C. After the mixture was homogeneously vortexed, propylene glycol and water were added, then homogeneously vortexed.	2.5 mg/g capsaicin, 63% glycerol monooleate, 7% propylene glycol 30%	Monooleate—solvent Propylene glycol, organic solvent decreases the cubic phase gel viscosity and it is a skin penetration enhancer.	Rate of release 33%	Cubic phase gels based on the ternary phase diagram of the monooleate–propylene glycol–water system offer transdermal controlled release of capsaicin. After 108 h, around 30% of capsaicin was released from the gels.	[72]
Capsaicin	Emulsion gel	Hot melt homogenization technique Capsaicin and melted lipids were thoroughly mixed using a magnetic stirrer, forming a clear lipid phase. A separate aqueous phase with Tween 80 was prepared. The hot lipid phase was then gradually added to the hot aqueous phase and homogenized.	Capsaicin, carbopol, ethanol, PEG400	Ethanol—solvent PEG400—solvent Carbopol, a rheology modifier and extended-release polymers that enhance the viscosity of gel and skin permeation and offers sustained release	Rate of release 59.2% Permeation rate (skin of Sprague–Dawley rats) 7.2% (after 24 h)	The capsaicin-loaded nanoparticles containing solution showed a higher analgesic efficacy than the capsaicin-loaded nanoparticles containing gel in the Hot-plate test. The capsaicin-based nanoparticles formulations presented increased analgesic efficacy than capsaicin cream at the same concentration in the Hot-plate test due to the higher permeation ability and improved skin retention. The gel induced slightly less irritation than the solutions.	[73]
Capsaicin	Biogel	Passive loading technique Capsaicin was dissolved in methanol using a sonicator. After adding natural β-chitin–protein complex film, the mix was vortexed at 120 rpm and incubated in a shaker at room temperature for 48 h.	100 mg capsaicin, 8 mL methanol, 200 mg β-chitin–protein complex	Methanol—solvent β-chitin–protein complex—natural polymer β-chitin–protein complex film has a finer structure in contrast to synthetically made chitosan films.	Rate of release pH 4.0: 50.49% (48 h) pH 5.5: 59.81% (72 h) pH 7.4: 59.02% (96 h)	β-chitin–protein complex film offers a prolonged release rate of capsaicin The maximum capsaicin release was at pH 7.4, which is slightly higher than that of the skin, after 96 h.	[74]
Capsaicin	Hydrogel	Interfacial deposition of preformed polymer method An organic phase containing Eudragit RS100^®^, capsaicinoids mixture, acetone, and capric/caprylic triglyceride was injected in an aqueous phase containing polysorbate 80. After mixing, the solvents were removed with a reduced-pressure evaporator.	5 mg capsaicinoids mixture, 3.5% chitosan, 1.5% lactic acid, 100 mg Eudragit RS100^®^	Lactic acid-pH modifier Eudragit RS100^®^-polymer Chitosan, a polysaccharide, that presents enhanced skin bioadhesion, film-forming capability, and wound-healing promotion	Rate of release 81 ± 1% (96 h)	The best formula had the lowest quantity of lactic acid, which provides a tolerable pH value (4.34 ± 0.11) and good biocompatibility.	[68]
Capsaicin	Emulsion gels	High shear mixing Capsaicin was added to a melted mixture of phytantriol and poloxamer 407 at 60 °C. This mixture was stirred with water, and after equilibrating for 48 h at room temperature, a cubic phase gel formed.	10.88 mg capsaicin, 0.3047 g PL F127, 3.0096 g phytantriol.	Phytantriol—solvent	Rate of release F1: 41% F2: 33% Permeation rate (skin of Sprague–Dawley rats) F1: 0.32 ± 0.05 μg·cm^−1^·h^−1^ F2: 0.18 ± 0.02 μg·cm^−2^·h^−1^	Phytantriol forms cubosomes determining a sustained release of capsaicine (longer than that of on-market products). Cubosomes formulations induced no obvious irritation to the skin.	[75]
Tramadol	Organogel	Coacervation phase separation method Proniosome components and tramadol were mixed with absolute ethyl alcohol and heated in a thermostatic water bath. Further, phosphate buffer (pH 7.4) was added on the water bath. After cooling down to room temperature, the solutions was mixed with HPMC.	100 mg tramadol, 1800 mg Span 80, 1800 mg lecithin, 100 mg cholesterol	Lecithin—nonionic surfactant Cholesterol—nonionic surfactant Span 80—hydrophobic surfactant	Rate of release 60% (6 h) Permeation rate (skin of New Zealand Wistar albino rats) 2300 µg/cm^−1^ (in 24 h)	The tramadol gel demonstrated higher analgesic efficacy than oral tablets in rat tests and showed significant anti-inflammatory effects. This formula had improved entrapment efficiency and transdermal flux compared to tramadol cream. The low transition temperature of Span80 contributed to a fluid state, facilitating drug transport into the skin.	[18]
Tramadol	Hydrogel	Irradiation with electron beams Tramadol solution was created by diluting tramadol injection in purified water. HPMC hydrogel films were purified and dried before being turned into xerogels. The xerogels were then submerged in tramadol solution for 24 h to obtain hydrogel films containing tramadol (30 or 50 kGy dose).	20% tramadol, 20% hydroxypropyl methylcellulose	HPMC—cellulosic polymer HPMC interaction with the drug is minimum and do not inhibit the sustained release of the drug HPMC produced a transparent film	Rate of release 100% (after 240 min) Permeation rate (skin of mice) 1.36 mg/cm^2^ (in 240 min)	The tramadol-containing gel showed a higher analgesic efficacy than oral tramadol tablets in acetic-acid-induced abdominal writhing test in rats. The new formula presented an enhanced release and skin permeation compared to standard formulations. The amount of tramadol released from the hydrogel film and the amount of skin permeation increased by changing the electron dose.	[78]
Tramadol	Organogel	Cold method The aqueous phase was heated at 30 °C and then delivered to the organic phase dropwise with constant mixing. The stir was continued for 45 min to bring the transethosomal dispersions that were supplemented for size reduction by probe sonication.	100 mg tramadol, 3% α phosphatidylcholine from egg yolk, 0.5% cremophor EL-35	α phosphatidylcholine—lipid carrier; showed low viscosity Cremophor EL-35—edge activator	Rate of release 79.98% (8 h)	The new formula exhibited a controlled rate of release compared to standard formulations. The use of edge activator in formulation increased the skin permeability.	[79]
Tramadol	Hydrogel	Dispersion Chitosan was dispersed in an acetic acid solution, and tramadol and poloxamer F-127 were dissolved in it. The mixture was placed in an ice bath for 30 min, then glycerophosphate disodium salt hydrate and pentasodium triphosphate were added. Finally, the mixture was moved to a 37 °C water bath, where gel formation occurred after about 1–1.5 min.	20% tramadol, 1% chitosan, 20% PL F-127, 14.5% glycerophosphate disodium salt hydrate, 0.5% pentasodium triphosphate	Chitosan, a natural polysaccharide that can form gels by interacting with other polymers or through physical and chemical cross-linking. It can form hydrogels by swelling in water.	Rate of release 80% (8 h)	Chitosan extends the release of drug compared to tramadol-containing gel.	[83]
Tramadol	Hydrogel	Cold method Tramadol was dispersed in various solutions containing PL 407 alone or in binary systems with PL 188 and were retained at 4 °C under magnetic stirring. The PL concentrations were selected in order to obtain a thermoreversible gel at minimum possible final concentration with a maximum final PL concentration of 35% (weight per weight [*w*/*w*]).	20 mg × mL ^−1^ tramadol, 25% PL 407, 10% PL 188	PL 407 and PL 188—nonionic surfactants	Rate of release 100% (after 4 h)	PL 407 and PL 188-based binary hydrogels showed controlled release of tramadol. Subsequently, they had extended duration of analgesia (72 h) compared to tramadol solution in tail-flick test. This leads to the possibility of reapplying every 48–72 h at lower doses. The gel showed an enhanced release rate at pH 7.4 and 37 °C.	[82]
Gabapentin	Hydrogel	Dissolution Gabapentin was dissolved into de-ionized water, and methyl and propyl hydroxybenzoate were dissolved in a permeation enhancer solvent. The enhancer mixture was added to the aqueous mixture and mixed for 5 min.	6% gabapentin, 0.75% carbopol	Carbopol—synthetic polymer carbomer	Mean flux value 2661.62 ± 50.39 Permeation rate (“nonhy-drated” human epidermal membrane) 7.56 ± 5.50 mcg/cm^2^/h	Carbopol can incorporate low-molecular-weight compounds, such as gabapentin. Prehydrated and non-prehydrated membranes had similar gabapentin permeability, but prehydrated ones showed less variability.	[88]
Gabapentin	Emulsion gel	-	10% gabapentin, xanthan gum hydrocolloid, polyacrylamide	-	-	A 10% *w*/*w* topical gabapentin gel applied thrice daily demonstrated strong antiallodynic and antihyperalgic effects in Hot-plate and von Frey test. Topical use of the gel potentially avoids dose titration in neuropathic pain patients, reduces pain similarly to systemic gabapentin, and avoids related side effects.	[86]
Gabapentin	Emulsion gel	-	10% gabapentin, xanthan gum hydrocolloid, polyacrylamide	-	-	Topical application of gabapentin highly reduced cisplatin-associated neuropathic allodynia and heat-hypoalgesia. It might offer an alternative for neuropathic pain relief in patients treated with chemotherapy or those intolerant to systemic medications’ side effects.	[87]
Gabapentin	Hydrogel	Copolymerization The chitosan-g-poly(acrylic acid-co-acrylamide) hydrogel was obtained following the reaction between a radical initiator (ammonium persulfate) and a cross-linking agent (N,N′-methylene bisacrylamide).	Gabapentin, acrylic acid, acrylamide monomers, chitosan	Chitosan—natural polymer Acrylamide monomers—cross-linkers	Rate of release 90% in the first 2 h	Acrylic acid and acrylamide monomers overcome the disadvantages of chitosan by cross-linking. In vitro simulation of gabapentin release from hydrogel in stomach-like (pH 1.2) and physiological buffer (pH 7.4) conditions showed direct drug diffusion from loaded gel samples in both pHs, with faster diffusion in pH 1.2 due to swelled samples.	[89]
Pregabalin	Organogel	Mixing A 1% aqueous solution of pregabalin was mixed with propylene glycol. This solution was then mixed with the oil phase solution of a PLO gel kit.	0.4% pregabalin, lecithin, isopropyl palmitate, PL 407	PL 407—block copolymer Lecithin—absorption enhancer Isopropyl palmitate—solvent for lecithin	Permeation rate (skin of mice) 0.7 µg/mL (after 120 h)	A significant analgesic effect was observed 1.5 h after application using von Frey test in mice streptozotocin-induced diabetic neuropathy.	[94]
Pregabalin	Hydrogel	High shear mixing A transparent gel was obtained by spraying with continuous stirring on the water surface 2% *w*/*w* gel bases (HPMC and carbopol 934). Then a uniform and clear solution was formed under continuous stirring by dissolving the pregabalin in the polymer dispersion.	5 g niosomes (1.5 g pregabalin F1 Span 60: cholesterol, 4:1 F2 Span 60: cholesterol, 4:4 F3 Span 60: cholesterol, 4:7) in HPMC or carbopol	Cholesterol enhanced the entrapment efficiency. Span 60, nonionic surfactant, gave the highest entrapment efficiency and stability of niosomes.	Release rate 32.2 ± 0.02% Permeation rate (skin of rats) 28.34% (in a period of 8 h)	-	[92]
Pregabalin	Hydrogel	Photopolymerization under both UV lights The pH-responsive layer was prepared using poly(methacrylic acid-g-ethylene glycol) as the macromer, eosin Y as the photoinitiator, and triethanolamine as the co-initiator. Hydrophobic domains were added by incorporating cross-linked styrene-butadiene-styrene (SBS) 30 copolymer in the pH-sensitive prepolymer.	3.6 g pregabalin, 2.0 g Poly(ethylene glycol) monomethyl ether monomethacrylate	-	Rate of release 86.4% at neutral pH Permeation rate (human fibroblast cell line): 28.34% over a period of 8 h	The gel was swollen and transparent at pH 7.0, while opaque at pH 2.2. Hydrogels formed with UV and visible light showed reversibility of swelling, responding to repeated pH variations in both low (pH 2.2) and high (pH 7.0) buffers.	[95]
Pregabalin	Emulsion gel	High shear mixing Span 80 was dissolved in castor oil (oil phase). Pregabalin and tween 80 were dissolved in distilled water, mixed with methyl and propyl parabens dissolved in propylene glycol (aqueous phase). Both phases were heated separately to 70–80 °C, before combining the oil phase with the aqueous phase.	2 g pregabalin, 10 g propylene glycol, 4 g Tween 80, 1 g Span 80, 10 g castor oil, 0.4 g carbopol 940.	Carbopol—polymer	Rate of release 30% in 20 min and 93% in 360 min	Carbopol in the specified amount resulted in the best drug release rate.	[93]
Amitriptyline	Organogel	High shear mixing Amitriptyline was mixed with Poloxamer 30% after being dissolved in sterile water. Isopropyl lecithin was mixed with the poloxamer component.	5% amitryptiline, 30% PL, lecithin-isopropyl myristate	-	-	No significant change in pain intensity vs. topical lidocaine in patients with postherpetic neuralgia, chronic postsurgical pain, and painful peripheral neuropathy	[97]
Amitriptyline	Organogel	High shear mixing Amitriptyline was finely powdered and mixed with ethoxy diglycol to obtain a smooth paste. Mediflo PLO gel was added.	10% amitryptiline, ethoxy diglycol, Mediflo PLO gel	-	Rate of release 53.2% Permeation rate (Strat-M membrane) 9.3% (over 24 h)	Mediflo PLO gel resulted in the highest release rate at 32 ± 0.5 °C and pH 7.4. However, Lipoderm base and Emollient Cream resulted in a lower cumulative permeation relative to Mediflo PLO gel.	[98]
Ketamine	Hydrogel	High shear mixing Ketamine was added in the Pluronic^®^ F127 stabilized reduced graphene oxide dispersion and kept under mild stirring for 24 h. The viscosity of the ketamine-loaded Pluronic^®^ F127 stabilized reduced graphene oxide dispersion was increased by adding 2% Carbopol 94 for topical application.	5% ketamine, 0.1 μg/mL pluronic F127 stabilized reduced graphene oxide, 2% Carbopol 940	Pluronic F127 stabilized reduced graphene oxide offered a prolonged release of ketamine due to the unique π-π stacking interaction between ketamine and reduced graphene oxide.	Permeation rate 120.0 μg/cm^2^/h	Ketamine-loaded reduced graphene oxide demonstrated sustained analgesic effect (24 h) compared to control hydrogel (4 h) in the tail-flick study. This alternative for neuropathic pain treatment using Pluronic^®^ F127 graphene oxide hydrogel avoids side effects and skin irritation associated with other administration routes.	[52]
Baclofen	Organogel	Thin-film hydration method	5% baclofen niosomes (baclofen/Span 60/40/cholesterol) in 1% Carbopol 934	Span60/40—nonionic surfactants Cholesterol, enhancer of niosomal membrane rigidity.	Rate of release 62.75% (in 24 h)–for niosomes Permeation rate (cellulose membrane) 99.51% (in 24 h) at a maintained temperature of 37 ± 0.5 °C and pH 5.5	Reduced carrageenan-induced paw edema similar to oral marketed tablets. Topically applied niosomes improve the residence time of drugs in the stratum corneum and epidermis, while decreasing the systemic absorption of drug Carbopol prints faster permeation to the skin. Niosomes offer a prolonged and controlled release of the drug.	[100]
Baclofen	Hydrogel	High shear mixing	F13: baclofen in Carbopol 934 (1:4) F18: baclofen in xanthan (1:6)	Carbopol/Xanthan-thickening agent, stabilizer, controlled release	Rate of release F13: 98.98% (after 24 h) Permeation rate (skin of Albino rats) F13: 4.27 µg/cm^2^ /h	Carbopol and xanthan as gelling agents showed enhanced permeation through the skin and release rate.	[101]
Naproxen	Inorganic gel	Dissolution Gantrez AN 119 and glycofurol were homogenized to form a clear dispersion, which was degassed under vacuum and stored at room temperature for 1 day before use. Naproxen was dissolved before the addition of the polymer.	5% naproxen, 2.5% Gantrez, glycofurol	Gantrez—copolymer, gelling and bioadhesive agent. Glycofurol—good adhesiveness and spreadability.	Rate of release 584.78 ± 32.8 μg/cm^2^ Permeation rate (skin of Sprague–Dawley rats) 161.168 ± 29.2 μg/cm^2^/h	No skin irritation Glycofurol use results in high drug permeation.	[104]
Naproxen	Hydrogel	Synthetic procedures	Naproxen, diphenylalanine, N-hydroxysuccinimide	Diphenylalanine-D-amino acid	Rate of release 35.8% after 24 h	Minimal adverse effects	[105]
Ibuprofen	Hydrogel	High shear mixing The clear microemulsion-based hydrogel was prepared by completely dissolving the xanthan gum in the microemulsion under stirring.	3% ibuprofen, 3% ethyl oleate, 20% Tween 80, 10% propylene glycol, 1.5% xanthan gum	Xanthan gum increases viscosity. Ethyl oleate enhances the solubilizing capacity of microemulsion systems. Tween 80 acts as a surfactant, and propylene glycol as a cosurfactant.	Rate of release 38.06 ± 1.04 (µg × cm^−2^ h^−1^) Permeation rate (porcine ear skin) 7.61 ± 0.21 × 10^−3^ cm × h^−1^	Ethyl oleate determines excellent skin permeation rate of ibuprofen.	[107]
Ibuprofen	Hydrogel	Dissolution Carbomer 974P was dissolved in a phosphate-buffered saline solution and pH was adjusted to 7.8 with 1 M NaOH. Agarose was added and the mixture was microwave-irradiated (500 W) for 30 s at 80 °C to initiate the condensation reaction. After cooling to 55 °C, the mixture was poured into steel cylindrical molds for gelation.	2.8% ibuprofen in carbomer 974P	-	Rate of release 80% after 48 h	Rate of release was pH-sensitive: at acidic or basic pH levels, increased hydrolytic rate was reported, with the alkaline conditions showing the fastest release (+10% cumulative release in acidic medium and +20% cumulative release in alkaline medium at 24 h, compared to neutral pH)-	[108]
Ibuprofen	Hydrogel	Freeze–thaw cycle Ibuprofen was dissolved in sodium hydroxide and polyethylene glycol. Chitosan was dissolved in 2% acetic acid and brought to 20 mL with distilled water to give a 2% *w*/*v* concentration. The drug–polymer suspension was obtained by adding the Ibuprofen solution to the chitosan solution. A nanoconjugate with a gum arabic gel matrix was formed by dispersing gum arabic propylene glycol solution and polyvinylpyrrolidone under continuous stirring and then incorporating the ibuprofen nanoconjugate into the gum arabic matrix solution.	50 mg ibuprofen, 4% phospholipon 90G, 2% chitosan, 10% polyvinyl alcohol, 2.5% gum arabic	Gum arabic—reduced the crystallinity of ibuprofen	Rate of release 90% (after approximately 13 h)	Nanoconjugate hydrogel ibuprofen-loaded chitosan–PC90G showed sustained and controlled release, surpassing the disadvantages associated with the oral dosage form.	[109]
Ibuprofen/Diclofenac	Inorganic gel	Dissolution Polymerization Ethyleneglycol dimethacrylate, N-(3-aminopropyl) methacrylamide, and 4-vinyl-pyridine were dissolved in 2-Hydroxyethyl methacrylate. The initiator 2, 20-azobis(isobutyronitrile) was added, and the monomer solution was injected into a mold. Polymerization occurred for 12 h at 50 °C followed by 24 h at 70 °C. The gels were then submerged in boiling water for 15 min to remove unreacted monomers. The resulting 10.5 mm diameter discs were washed in water, 0.9% NaCl, and 0.1 M HCl, and finally dried at 40 °C.	5 mg/g diclofenac, 18 mg/g ibuprofen, 4-vinyl-pyridine, N-(3-aminopropyl) methacrylamide, ethyleneglycol dimethacrylate	4-vinyl-pyridine and N-(3-aminopropyl) methacrylamide remarkably increased the amount of ibuprofen and diclofenac loaded.	Rate of release 60% for both ibuprofen and diclofenac Sustained release for 24 h for ibuprofen Sustained release for 1 week for diclofenac.	No difference was observed in the release rate at both pH 5.8 and 8.0.	[110]
Ibuprofen	Biogel	Layer-by-layer self-assembly method The chitosan hydrochloride and sodium cellulose sulfate were dissolved in distilled water and ultrasonized. This mixture was used as the intermediate layer solution. Aqueous solutions of 2% (*w*/*v*) sodium cellulose sulfate and 2% (*w*/*v*) sodium tripolyphosphate were used as the upper and lower layer solutions, respectively.	5% ibuprofen 2% sodium tripolyphosphate in chitosan hydrochloride/sodium cellulose sulfate (4:1)	Sodium cellulose sulfate—polyanionic polymer chitosan—natural polymer	Rate of release 27.2 ± 1.0% (in the first 60 min) 35.3 ± 1.88% (after 1440 min) Permeation rate (skin of mice) 3140.44 ± 159.89 mg/cm (24 h)	Sodium cellulose sulfate, chitosan hydrochloride, and sodium tripolyphosphate determine a favorable sustained release profile, no cytotoxicity, and good biocompatibility.	[39]
Baclofen, amitriptyline, ketamine	Organogel	-	10 mg baclofen, 40 mg amitriptyline, 20 mg ketamine in PL lecithin	-	-	Double-blind, placebo-controlled trial (chemotherapy-induced peripheral neuropathy) Slight improvement in sensory pain and motor scale vs. placebo Topical gel was well tolerated, without evident systemic toxicity	[96]
Baclofen, amitriptyline, ketamine	Organogel	-	10 mg baclofen, 40 mg amitriptyline, and 20 mg ketamine in PL lecithin	-	-	Prospective, single-arm, cohort pilot study (neuropathic pain caused by radiation skin reaction): reduction in pain intensity, sharpness, burning, sensitivity, itchiness, and unpleasantness, at 30 min posttreatment and at 2 weeks posttreatment. The gel may prove effective in relieving pain in subjects who do not respond to standard treatment, such as opioids. The efficacy of gel in reducing burning pain may be explained by blocking the release of chemical mediators or stimulation of ion channels by drugs, such as amitriptyline, ketamine	[111]
Amitriptyline, ketamine	-	-	2% amitriptyline and 0.5% ketamine in methylcellulose	-	-	Case report (patient with erythromelalgia): topical gel decreased the intensity of pain, and enhanced the functional status and quality of life of a patient (which had not responded satisfactory to oral paracetamol, NSAIDs, gabapentin, and amitriptyline). The gel made it possible to stop all other pain medications of patient. Due to biochemical effects of amitriptyline and ketamine, the gel could avert vasodilation and decrease the redness and high skin temperature characteristic of this disorder.	[112]

Legend: CMC-Na, sodium carboxymethyl cellulose; COX, cytochrome c oxidase; DMSO, dimethyl sulfoxide; EDTA, ethylenediaminetetraacetic acid; HEPES, 4-1-piperazineethanesulfonic acid; HPMC, hydroxypropyl methylcellulose; NSAID, nonsteroidal anti-inflammatory drugs; PEG400, polyethylene glycol 400; PL188, poloxamer 188; PL407, poloxamer 407; PLO, pluronic lecithin organogel; P(MAA625 g-EG), poly(methacrylic acid-g-ethylene glycol; SBS, styrene–butadiene–styrene block copolymer; UV light, ultraviolet light.

## Data Availability

All data generated or analyzed during this study are included in this published article.
